# Elevated CO_2_ Priming as a Sustainable Approach to Increasing Rice Tiller Number and Yield Potential

**DOI:** 10.1186/s12284-023-00629-0

**Published:** 2023-03-22

**Authors:** Jennifer M. Sloan, Azzami Adam Muhamad Mujab, Jusoh Mashitah, Berahim Zulkarami, Matthew J. Wilson, Liang Su Toh, A. Jalil Nur Zahirah, Kamaruzali Afiq, Ahmad Tajuddin Asyraf, Xin-Guang Zhu, Nazmin Yaapar, Andrew J. Fleming

**Affiliations:** 1 School of Biosciences, Plants, Photosynthesis and Soil, The University of Sheffield, Western Bank, Sheffield, S10 2TN UK; 2 Commercialization and Business Centre, Malaysian Agricultural Research and Development Institute, MARDI Parit, 32800 Parit, Perak Malaysia; 3 Department of Crop Science, Faculty of Agriculture, Universiti Putra Malaysia, UPM, 43400 Serdang, Selangor Malaysia; 4 Institute of Tropical Agriculture and Food Security, Universiti Putra Malaysia, UPM, 43400 Serdang, Selangor Malaysia; 5 Center of Excellence for Molecular Plant Science, Institute of Plant Physiology and Ecology, CAS, Shanghai, 200032 China

**Keywords:** Tiller, Yield, CO_2_, Climate, Rice

## Abstract

Tillering and yield are linked in rice, with significant efforts being invested to understand the genetic basis of this phenomenon. However, in addition to genetic factors, tillering is also influenced by the environment. Exploiting experiments in which seedlings were first grown in elevated CO_2_ (eCO_2_) before transfer and further growth under ambient CO_2_ (aCO_2_) levels, we found that even moderate exposure times to eCO_2_ were sufficient to induce tillering in seedlings, which was maintained in plants grown to maturity plants in controlled environment chambers. We then explored whether brief exposure to eCO_2_ (eCO_2_ priming) could be implemented to regulate tiller number and yield in the field. We designed a cost-effective growth system, using yeast to increase the CO_2_ level for the first 24 days of growth, and grew these seedlings to maturity in semi-field conditions in Malaysia. The increased growth caused by eCO_2_ priming translated into larger mature plants with increased tillering, panicle number, and improved grain filling and 1000 grain weight. In order to make the process more appealing to conventional rice farmers, we then developed a system in which fungal mycelium was used to generate the eCO_2_ via respiration of sugars derived by growing the fungus on lignocellulosic waste. Not only does this provide a sustainable source of CO_2_, it also has the added financial benefit to farmers of generating economically valuable oyster mushrooms as an end-product of mycelium growth. Our experiments show that the system is capable of generating sufficient CO_2_ to induce increased tillering in rice seedlings, leading eventually to 18% more tillers and panicles in mature paddy-grown crop. We discuss the potential of eCO_2_ priming as a rapidly implementable, broadly applicable and sustainable system to increase tillering, and thus yield potential in rice.

**Supplementary Information:**

The online version contains supplementary material available at 10.1186/s12284-023-00629-0.

## Background

Global annual increases in rice production are decreasing at a time of an increasing global population dependent on this crop as a staple food (Bin Rahman and Zhang 2022). This has led to an array of efforts to increase rice yield, both by breeding and implementing new agronomic approaches (Saito et al. 2021; Verma et al. 2021). Increased yields must be achieved in an environmentally and economically sustainable fashion—inputs to the agronomic system must be maintained at as low a level as feasible, and there must be economic incentives for rice farmers. For example, in Malaysia, the site of the experiments reported here, rice farmers represent an aging population whose income falls into the lower socio-economic bracket, despite the importance of rice nutritionally, socially and culturally to the wider society (Department of Statistics Malaysia 2021a, b).

With respect to rice yield, tiller number is closely linked to panicle number and, thus, the capacity of a plant to produce seed (Huang et al. 2020). Consequently, there have been numerous studies into the genetic basis for tillering and panicle development in rice (Saad 2014; Liang et al. 2014; Zhao et al. 2020; Huang et al. 2021), with significant advances in the identification of genetic traits controlling tiller number and inflorescence architecture. Studies have also provided insight into the complex network of internal signaling processes controlling tiller formation and bud development, including, for example, sucrose (Patil et al. 2022) and strigolactone (Fang et al. 2020). However, in addition to genetic factors, it is clear that environmental factors can also influence tiller development (Wang and Li 2005; Assuero and Tognetti 2010; Saad 2014). These include the circadian clock (Wang et al. 2020), temperature (Xu et al. 2020), CO_2_ (Seneweera 2011; Zhou et al. 2021; Liu et al. 2021) and the availability of nutrients (Wang et al. 2017).

With respect to CO_2_, a meta-analysis of 20 years of rice Free Air CO_2_ Enrichment studies, showed that panicle number per ha, spikelet number per panicle, fully-filled grain percentage and grain weight were all significantly increased in plants grown at high levels of CO_2_ (500–645 ppm) throughout their lifetime (Hu et al. 2021). To what extent these results reflected a general increase in growth linked to prolonged exposure to CO_2_ or whether there was a more specific developmental response to elevated CO_2_ remains unclear. For example, Jitla et al. (1997) showed that exposure to high levels of CO_2_ increased the number of tillers in mature *Oryza sativa* L., and that rice plants can respond to elevated CO_2_ at an early stage of development, but further analysis of this response has been limited. It is interesting that some leaf traits, such as stomatal density, can be set extremely early in leaf development by CO_2_ level, with the phenotype of the mature leaf set during this early phase (Lake et al. 2001). It is unknown whether other aspects of plant structure such as tiller formation display a similar regulation, i.e., at what point in rice plant development is tiller number set?

In this paper, we first describe a series of experiments designed to explore when rice plants can respond to CO_2_ in terms of increased tillering. We then explore whether the observed response can be exploited to induce tillering and yield in semi-field and field conditions in Malaysia, a hub of rice production in S.E. Asia.

## Materials and Methods

### Plant Material and Growth Conditions

*Oryza sativa* (Indica-IR64) seeds were provided by the International Rice Research institute. *Oryza sativa* (Indica-MR219) and *Oryza sativa* (Indica-MR263) seeds were provided by the Genebank and Seed Centre, Malaysian Agricultural Research and Development Institute (MARDI).

### Commercial Controlled Environment Chambers

Experiments were carried out in a set of paired plant growth chambers (Conviron PGR15; Conviron, Winnipeg, Canada) at 70% relative humidity, in a 12/12 h light/dark cycle at 28/24 °C with a light intensity of 750 μmol m^−2^ s^−1^ at canopy height. CO_2_ was either kept at ambient levels (430 ppm) with no added CO_2_ (aCO_2_ chamber) or CO_2_ was controlled at 800 ppm (eCO_2_ chamber). Seeds were germinated on filter paper with 15 ml water in petri dishes, then grown in 13D pots (0.88L) filled with 71% Kettering Loam (Boughton, UK), 23.5% Vitax John Innes No. 3 (Leicester, UK), 5% silica sand and 0.5% Osmocote Extract Standard 5–6 month slow-release fertilizer (ICL, Ipswich, UK) by volume, saturated with water. aCO_2_ plants and eCO_2_ plants were germinated and grown exclusively in the aCO_2_ chamber or eCO_2_ chamber respectively. eCO_2_ primed plants were germinated and grown in the eCO_2_ chamber for the number of days indicated, then transferred to the aCO_2_ chamber. Ten plants were used for tillering analysis except for the aCO2 treatment of MR219 where n = 9.

### Yeast-Controlled Propagators—Semi-Field Trial

Propagators were constructed as shown in Additional file 1: Fig. S1A. Temperature was controlled at 24–30 °C, with a 12 h/12 h light/dark cycle. Light was provided by 12 units of 30 watts, 1.2 m T8 LED growth light with a red:blue LED ratio of 3:1, and one unit of white 16-W T5 LED. Relative humidity for aCO_2_ was 72–76% (day) and 73–83% (night), and for eCO_2_ 78–80% (day) and 80–86% (night). A mix of 600 g sugar, 1.5 L distilled water, and a packet of 11 g baker's yeast (*Saccharomyces cerevisiae*) in a 2.7 L plastic bottle was used to generate eCO_2_, with detailed measurements of CO_2_ shown in Additional file 1: Fig. S1B. Each fermentation mix lasted 10 days before being replaced. Seeds were soaked in distilled water for 24 h, then germinated in the aCO_2_ or eCO_2_ propagators on wet paper towel in a petri dish for 2–3 days, then three seedlings were transferred to each 8 fl oz container with standard soil media mixture (3:2:1 topsoil:sand:burned rice husk), and submerged in 2–3 cm of water. Seedlings were fertilized at day 5 and 15 with 1 g NPK 15:15:15 and 1 g urea per pot. 25 days after sowing (DAS), seedlings were transplanted two seedlings to a 1 gallon container filled with soil taken from a paddy field in Tanjong Karang, Selangor and transferred to semi-field conditions (a rainout shelter structure at Field 15 research farm, Universiti Putra Malaysia, Serdang) from October 2018 to March 2019. Plants were drip irrigated. Air temperature was 25–38 °C, humidity level was 60–80% and ambient CO_2_ ranged between 374 and 434 ppm. The fertilisation schedule was as in (Saad 2014). Wood vinegar and neem oil-based solution was applied every two weeks, and solar-powered ultrasonic animal repellent (OEM, China) were used to control pest infestations.

### Mycelium-Controlled Chambers—Paddy Trial

The chambers each contained a full spectrum LED light panel (Samsung LM281B AC 100–265 V, Shenzhen Colighting Ltd., China) and a six-inch exhaust fan (Additional file 2: Fig. S2A). A light/dark 11/13 h schedule was used, with light intensity measured using a spectrometer (LI-180; LI-COR Inc., Lincoln, NE, USA) at 740 μmol m^−2^ s^−1^ PPFD (photosynthetic photon flux density) (Blue mean intensity: 119 μmol m^−2^ s^−1^; Red mean intensity 308 μmol m^−2^ s^−1^). Oyster mushroom (*Pleurotus pulmonarius* (Fr.) Quel) mycelium bags used a substrate ratio of 100:10:1 sawdust:rice bran:CaCO_3_ powder and water (60–70% of sawdust weight) from Nas Agro Mushroom Farm, Malaysia. The eCO_2_ chamber contained 34 one-week injected mycelium bags, covered with a damp cloth. CO_2_ levels are shown in Additional file 2: Fig. S2B. Seeds were germinated within the aCO_2_ and eCO_2_ chambers for 3 days, then transplanted into a 12 pod tray (19 cm × 14.5 cm × 11 cm) filled with saturated peat moss:neem cake 10:1 and covered with a plastic propagator lid. Seedlings were thinned at 7 DAS to one seedling per pod. Neem oil (0.5% v/v) was sprayed at 14 DAS, 21 DAS and 28 DAS. The watering solution was changed to AB fertiliser solution with a concentration of 1800–2000 µS/cm at 8 DAS and was replaced every two days. At 30 DAS, seedlings were transplanted into 6 × 0.9 m^2^ irrigated plots, with a spacing of 30 cm between plants. The paddy soil was a silty clay texture (0.80% sand, 54.20% silt, and 45.10% clay) with pH 4.74 and 1.30% organic carbon. Plants were grown in the paddy field at the MARDI Field Station, Parit, Perak, from April-July 2022. Irrigation was maintained at 5–10 cm above the ground until 95 DAS. Insecticides (Karate—Syngenta, Match 050 EC—Syngenta, Nurelle D505 EC—Dow Agrosciences, Prevathon 5SC—DuPont, Pexalon—Dupont, Alika ZC—Syngenta), herbicides (Sofit 300 EC—Syngenta, Loyant—Dow Agrosciences), fungicide (Stinger—DuPont), and fertilisers (NPK Blue (12:12:17:2TE) and urea) were used following the typical schedules used at the MARDI Field Station.

### Plant Growth and Yield Analysis

A tiller was defined as any stem with more than one leaf—the main culm was not included in counts. The basal 5 cm of rice plants were prepared for tiller base images by fixing in 1:4 Acetic anhydride:EtOH for two weeks. The sample was cut to 1 cm above the base of the shoot and imaged from above on a LEICA M165 FC Stereomicroscope and built in LEICA DFC 450 C camera. Developing tillers were marked and counted.

Seedling measurements were taken at 24DAS for the yeast-controlled propagators, and 28DAS for the mycelium-controlled chamber. Measurement of plant height was taken from the surface of the soil to the highest shoot tip. Leaf number was manually counted on the fully expanded leaves. For plant height and leaf number measurements, each point is the mean of three seedlings. For dry weights, plants were separated into shoot and root, then dried for 48–72 h at 60C before weighing.

Sixteen plants were used in all semi-field yield analyses, except grain filling where a sample of 10 plants were used. Paddy-field yield analyses were performed on 29 or 30 plants, as indicated. Yield was analysed at 141 DAS (semi-field conditions) and 127 DAS (paddy conditions). Tillers and panicles were counted manually. Leaf biomass was dried as for seedling dry weights. Dried filled grains and unfilled and partially filled grains were counted. A complete set of 16 spikelets from 16 (semi-field experiment) or 29/30 (paddy-field experiment) were counted for 1000 grain weight using a seed counter (TRILITE, China). Percentage of filled seeds was calculated.

Semi-field yield was calculated as:

$$\begin{aligned} Yield\left( {g\;plant^{ - 1} } \right) = & Mean\;no.\;panicles \;per\;plant \times Mean\;no.\;spikelets\;per\;panicle \\ & \times \;\frac{1000\;g\;weight}{{1000}} \\ \end{aligned}$$

Paddy field yield was calculated as:

$$Yield\left( {tonne\;ha^{ - 1} } \right) = \frac{{\frac{Mean\;grain\;mass\;per\;plant \times no.\;plant}{{area\left( {m^{2} } \right) \times ha}}}}{1,000,000}$$

### Statistical Analysis

All statistical analysis was performed in Graphpad Prism versus 9.3.1.

## Results

### eCO_2_ Priming in Controlled Environment Chambers Leads to Increased Tillering in Rice

**Fig. 1:**
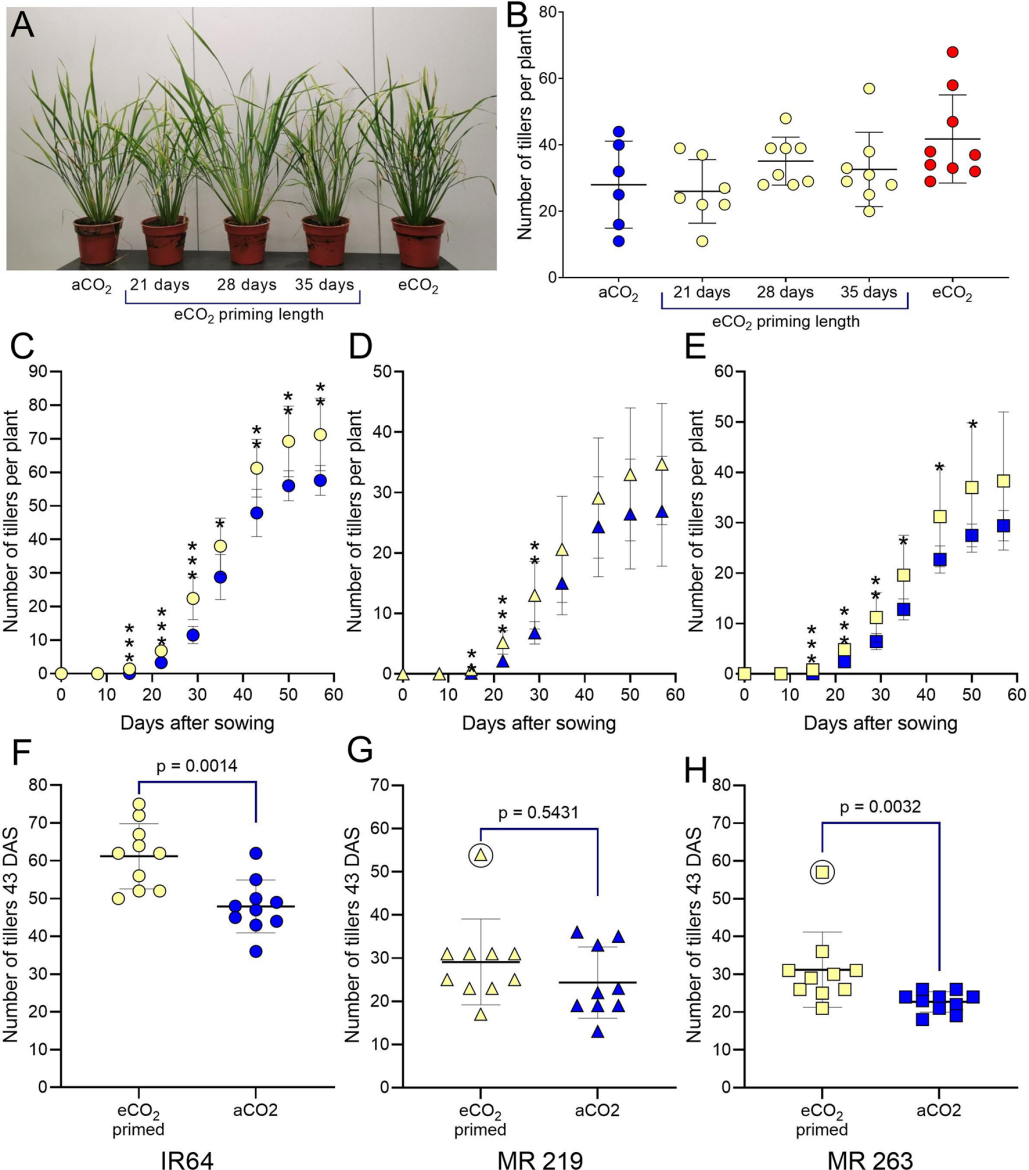
Phased exposure to eCO_2_ leads to increased tillering in rice. **A** Images of 49 day old IR64 grown in CE chambers. Plants were grown at either aCO_2_, or transferred to aCO_2_ from eCO_2_ after 21d, 28d, or 35d or grown exclusively at eCO_2_. **B** Number of tillers per plant at 49 DAS (n = 7/8). **C**, **F** IR64-circles, **D**, **G** MR219-triangles, **E**, **H** MR263-squares. **C**–**E** Number of tillers per plant over time. Yellow shapes represent plants eCO_2_-primed for 28 days, then transferred to aCO_2_. Blue shapes represent plants grown continuously at aCO_2_. **F**–**H** eCO_2_-primed IR64 plants (**F**) and MR263 plants (**H**) had more tillers at 43 days after sowing. MR219 plants **G** did not have a significantly different number of tillers after priming (unpaired t-test, *p* values as shown or *p < 0.05, **p < 0.01, ***p < 0.005, n = 9/10). Anomalous results (circled in G, H) are excluded from the statistical analysis

Rice plants grown constantly under elevated CO_2_ (eCO_2_) conditions showed an increased number of tillers (Fig. 1A, B). To identify when this increase was set, we grew young plants at high levels of CO_2_ for defined periods before transferring the plants to ambient CO_2_ levels, then measuring tiller number at plant maturity. Thus, rice plants (IR64) were primed at high CO_2_ for the first 21, 28 or 35 days after sowing (DAS) and then transferred to ambient CO_2_ (aCO_2_) conditions until they reached vegetative maturity (Fig. 1A, B). Control plants were maintained at aCO_2_ and eCO_2_ for the duration of the experiment. Plants which were eCO_2_ primed for 21 days were indistinguishable from aCO_2_ controls, however, plants eCO_2_ primed for 28 or 35 days had an increased number of tillers, comparable to the eCO_2_ controls at 49 DAS.

To better understand this increased tillering response, and to see if the effect was replicable with different cultivars, seedlings of IR64 and two cultivars of elite rice grown in Malaysia (MR219 and MR263) were eCO_2_ primed for 28 days then transferred to aCO_2_ conditions. These were compared to plants grown only under aCO_2_ conditions. Tillers were counted weekly from sowing to 57 DAS (Fig. 1C–H). All three cultivars showed a significantly increased number of tillers by 14 DAS. In IR64 and MR263 plants, the difference in tiller number remained significant until the end of the growth period (57 DAS) (unpaired t tests, * *p* < 0.05, ** *p* < 0.01, *** *p* < 0.005, n = 9/10) (Fig. 1C, E). Individual data points are shown from 43 DAS in Fig. 1F, H. MR219 plants had a higher mean number of tillers throughout the growth period, but this difference was not significant after 28 DAS (Fig. 1D, G). Plants grown in the same conditions were destructively sampled at 28 DAS to show the increased number of tillers at the base of the plant when grown at eCO_2_ (Additional file 3: Fig. S3).

### Yeast Driven eCO_2_ Priming Increases Seedling Growth and Final Yield

The results shown in Fig. 1 indicated that exposure of rice seedlings to eCO_2_ for 28 days led to increased tillering at maturity, a factor expected to lead to increased crop yield in the field. This time period is similar to that used in traditional rice cultivation where seedlings are first grown in nursery plots prior to transfer to the paddy. To explore whether our observations based on controlled environment chambers could be of potential value in an agronomic setting, we set out to replicate the CO_2_ priming treatment in a rice growing area in Malaysia.

**Fig. 2:**
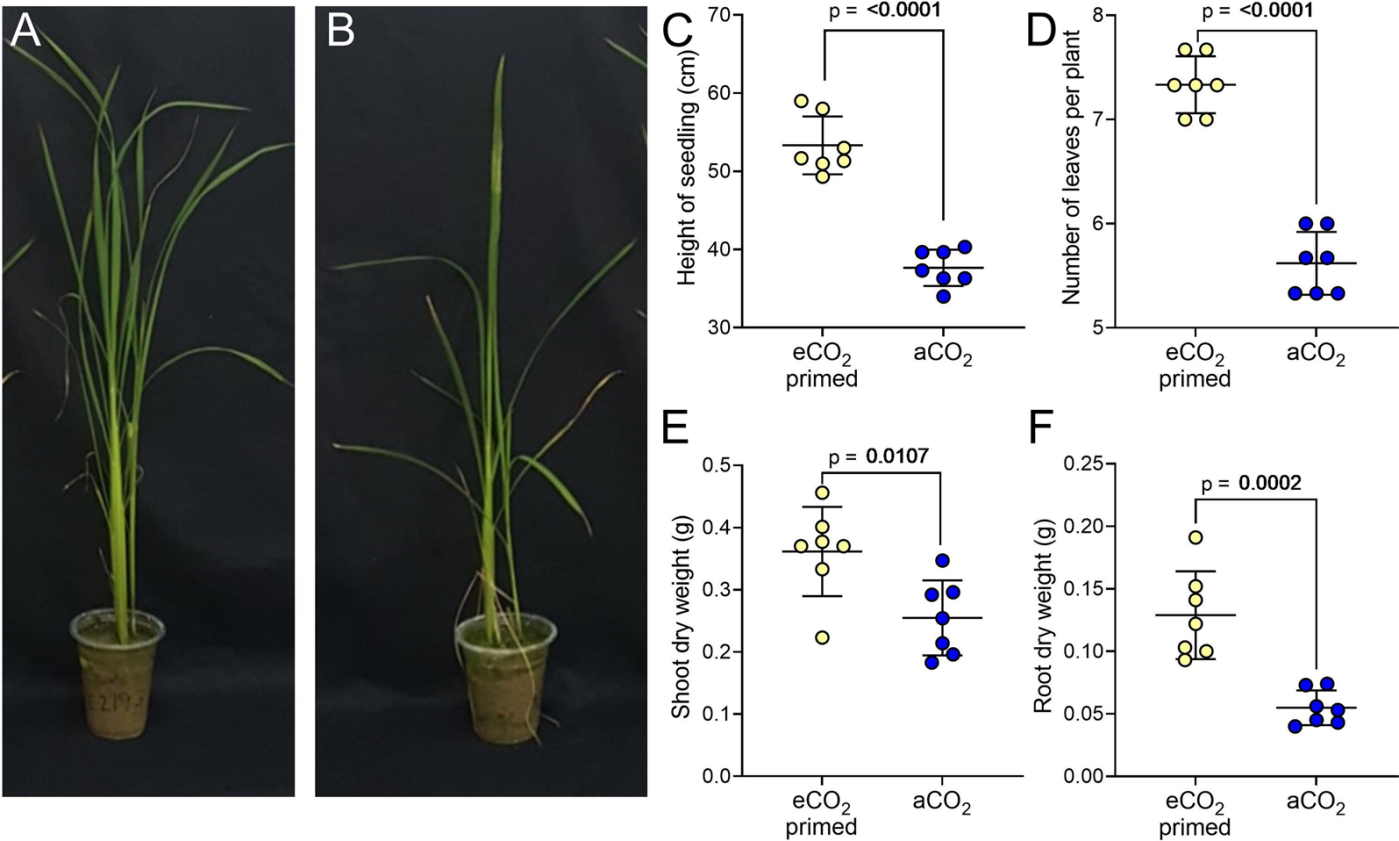
Rice seedling growth is increased in a yeast eCO_2_ propagator. MR219 seedlings 24 DAS grown **A** in a yeast eCO_2_ propagator and **B** at aCO_2_. **C**–**F** For seedlings harvested at 24 DAS, seedling height (**C**); leaf number (**D**); shoot dry weight (**E**) and root dry weight (**F**) are significantly higher in eCO_2_ grown plants. **C**–**F** unpaired t test, *p* values as shown, n = 7. For **C**, **D** each point is the mean of 3 seedlings

To do this, we first developed an economic in-house growth propagator system capable of creating elevated CO_2_ conditions using a combination of yeast, water and sugar to generate CO_2_ (Additional file 1: Fig. S1A). CO_2_ levels within the eCO_2_ propagator were on average 1000 ppm during the day and between 1500 and 2000 ppm at night, while the aCO_2_ propagator had reasonably constant CO_2_ levels of between 390 and 420 ppm (Additional file 1: Fig. S1A). We could thus generate a CO_2_-enriched environment for seedling growth. MR219 seedlings were grown in the eCO_2_ propagator for 24 days (Fig. 2A) and compared with plants grown for the same time in a propagator under aCO_2_ conditions (Fig. 2B). eCO_2_ grown plants were significantly taller, with an average of 42% increase in height compared with aCO_2_ controls (Fig. 2C, unpaired t test, *p* < 0.0001, n = 7). Seedlings grown at eCO_2_ also had more leaves than aCO_2_ controls (Fig. 2D, unpaired t test, *p* < 0.0001, n = 7), a 31% increase in shoot weight, and a 160% increase in root weight (Fig. 2E, F, unpaired t test, *p* = 0.0107 and *p* = 0.0002, n = 7). Thus, growing rice seedlings in the eCO_2_ propagator in Malaysia for a short time led to a promotion of growth.

**Fig. 3:**
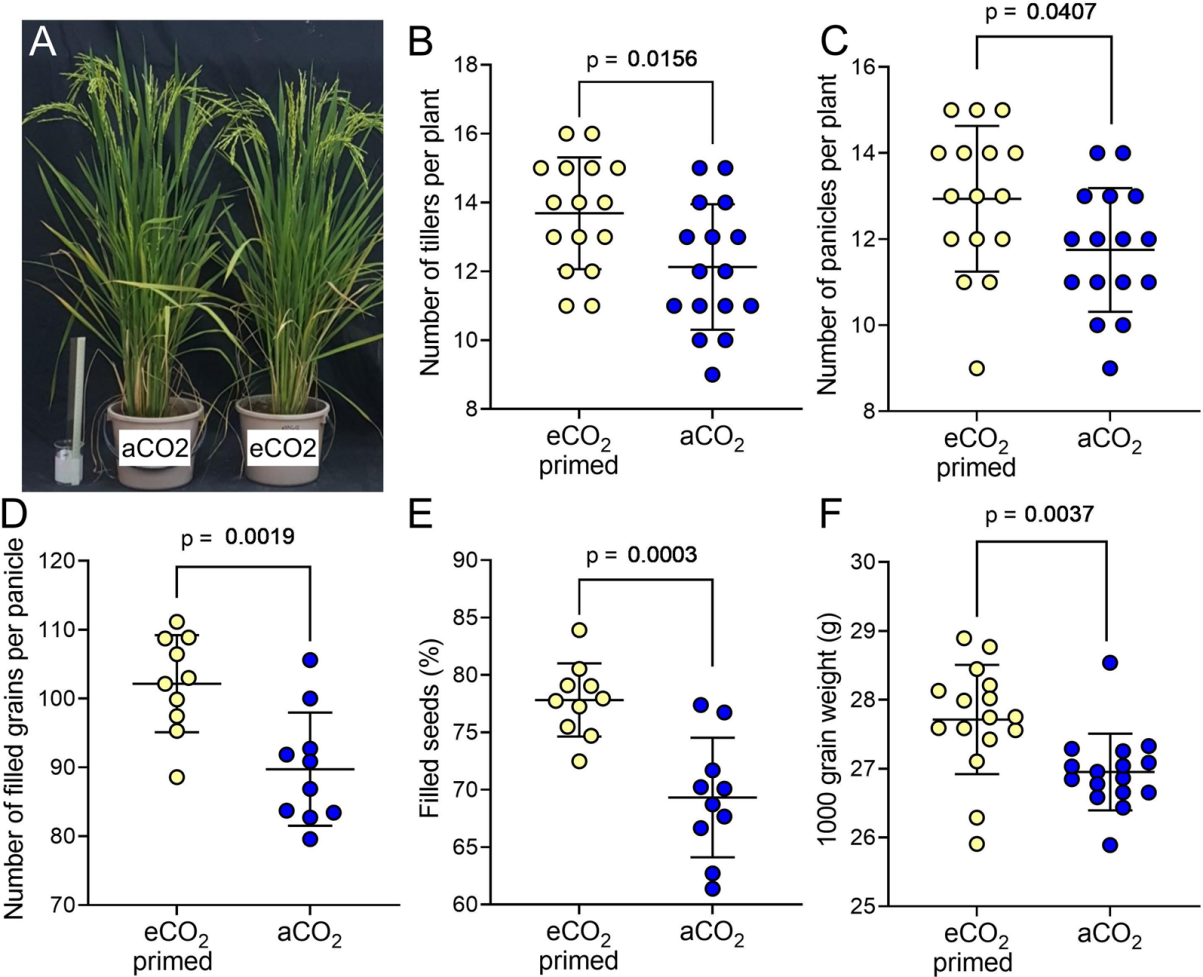
Yeast-derived eCO_2_ primed plants grown to maturity in semi-field conditions have increased growth and yield. **A** aCO_2_ and eCO_2_-primed MR219 plants at 90 DAS. **B**–**F** For plants harvested at 141 DAS, number of tillers per plant (**B**), number of panicles per plant (**C**), number of filled grains per panicle (**D**), the percentage of grains which are filled (**E**) and 1000 grain weight (**F**) are significantly higher in eCO_2_-primed plants. Unpaired t tests, *p* values as shown. **B**, **C**, **F** n = 16; **D**, **E** n = 10

**Fig. 4:**
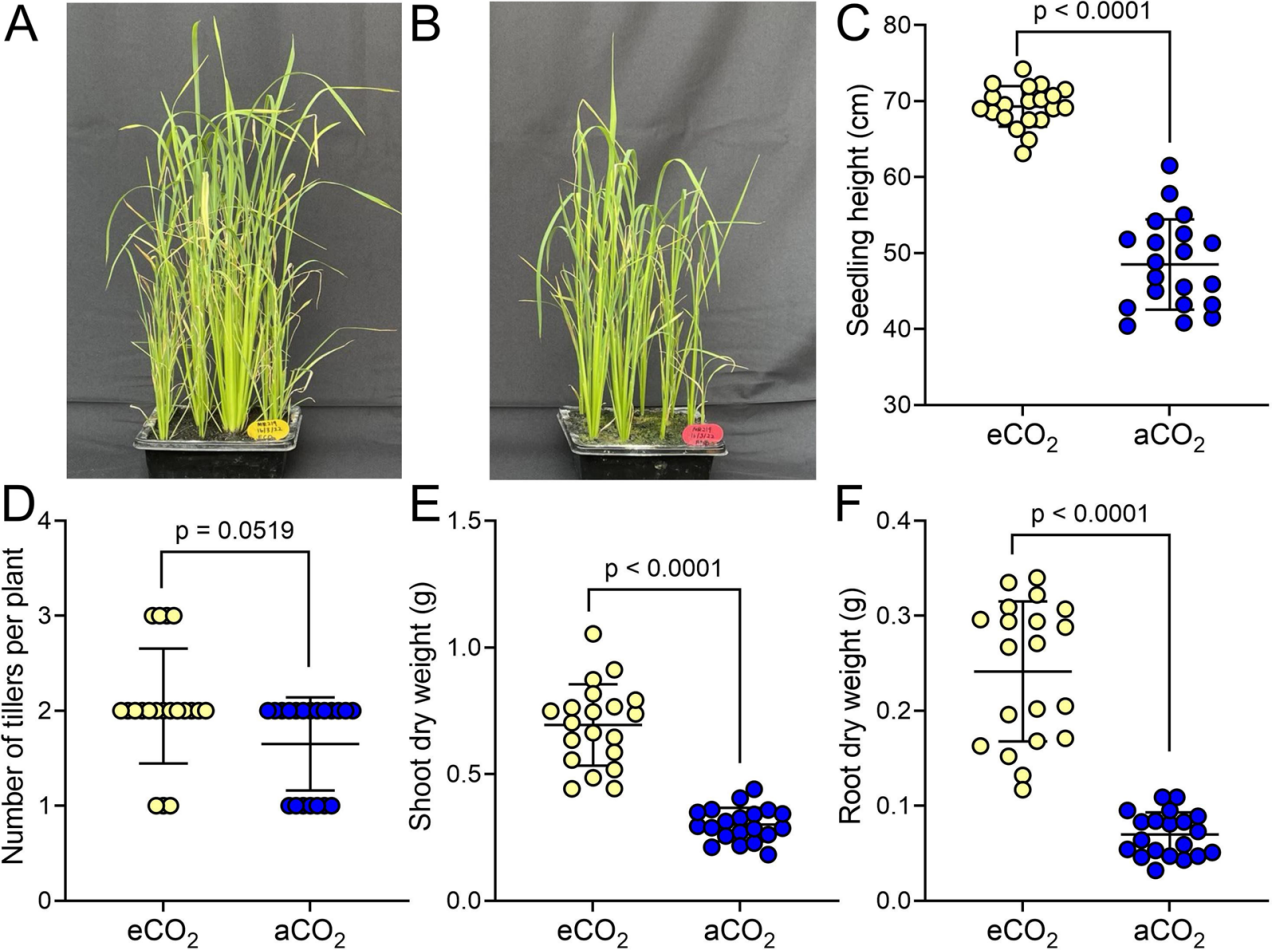
Rice seedling growth is increased in a mycelium eCO_2_ chamber. **A**, **B** MR219 seedlings 27 DAS grown at eCO_2_ in mycelium controlled chamber (**A**) or at aCO_2_ (**B**). **C**–**F** For seedlings harvested at 28 DAS, seedling height (**C**); number of tillers (**D**); shoot dry weight (**E**) and root dry weight (**F**) is higher in eCO_2_ grown seedlings. **C**, **E** Unpaired t test with Welch’s correction, n = 20; **D** Mann–Whitney U test, n = 20; **F** Unpaired t test, n = 20. *p* values as shown

To investigate the outcome of elevated CO_2_ during the seedling stage on final plant yield, a semi-field trial was performed in which eCO_2_ primed and aCO_2_ seedlings were transplanted into 1-gallon containers (two plants per container) and moved into a field-based rain shelter in a random block design until maturity (Fig. 3A). At 141 DAS, plants were harvested, and various parameters were measured (Fig. 3B–F). Plants which had been eCO_2_ primed in the yeast propagator for 25 days had more tillers (Fig. 3B) and panicles (Fig. 3C) than those grown at aCO_2_ for the duration of their growth. Analysis of yield on ten plants per treatment revealed that the number of filled grains per panicle was improved in eCO_2_ primed plants (Fig. 3D, unpaired t test, *p* = 0.0019, n = 10) as was the percentage of filled seeds (Fig. 3E, *p* = 0.0003, n = 10). Thousand grain weight was also increased in the eCO_2_ treated plants compared with the controls (Fig. 4F, unpaired t test, *p* = 0.0037, n = 16). Based on these data, an average yield per plant was calculated as 36.6 g for eCO_2_ primed plants, and 28.4 g for aCO_2_ control plants, equivalent to an estimated yield increase of 29%.

### Mycelium Generated eCO_2_ Priming Increases Plant Growth in Paddy Conditions

Encouraged by the semi-field experiments, we adapted the CO_2_ priming system to make it more applicable to the field. In order to find a system that not only elevated local CO_2_, but also provided a potential extra revenue source for farmers, we investigated using bags of Oyster mushroom fungal mycelium rather than yeast as a CO_2_ source. The chamber design was also improved to increase ease of use (Additional file 2: Fig. S2A). Oyster mushrooms were chosen since the fruiting bodies have relatively high economic value. Under our growth conditions, mycelium bags produced fruiting bodies after 28–35 days, with a second bloom after a further 20–25 days. In addition, the compost generated by mycelium breakdown has a market value for horticulture. Finally, the substrate used to grow the fungus was agricultural lignocellulosic waste, which is both plentiful and low-cost in Malaysia. Under our conditions, the mycelium-derived CO_2_ showed a diurnal cycle, with an average CO_2_ level of 655 ppm during the day and 1540 ppm at night in the eCO_2_ chamber. In contrast the aCO_2_ chamber showed a fairly constant level of 450 ppm CO_2_ (Additional file 2: Fig. S2B).

The effect of 28 days of mycelium-driven eCO_2_ priming on rice seedlings was similar to that observed in plants grown in the yeast eCO_2_ propagator (Fig. 4A, B). eCO_2_ primed seedlings were taller than aCO_2_ controls (Fig. 4C, unpaired t test with Welch’s correction, *p* < 0.0001, n = 20) and had a slightly increased number of tillers (Fig. 4D, Mann–Whitney U test, *p* = 0.0519, n = 20). There was an increased shoot weight (Fig. 4E, unpaired t test with Welch’s correction, *p* < 0.0001, n = 20) and root weight (Fig. 4E, unpaired t test, *p* < 0.0001, n = 20) in the eCO_2_ plants.

**Fig. 5:**
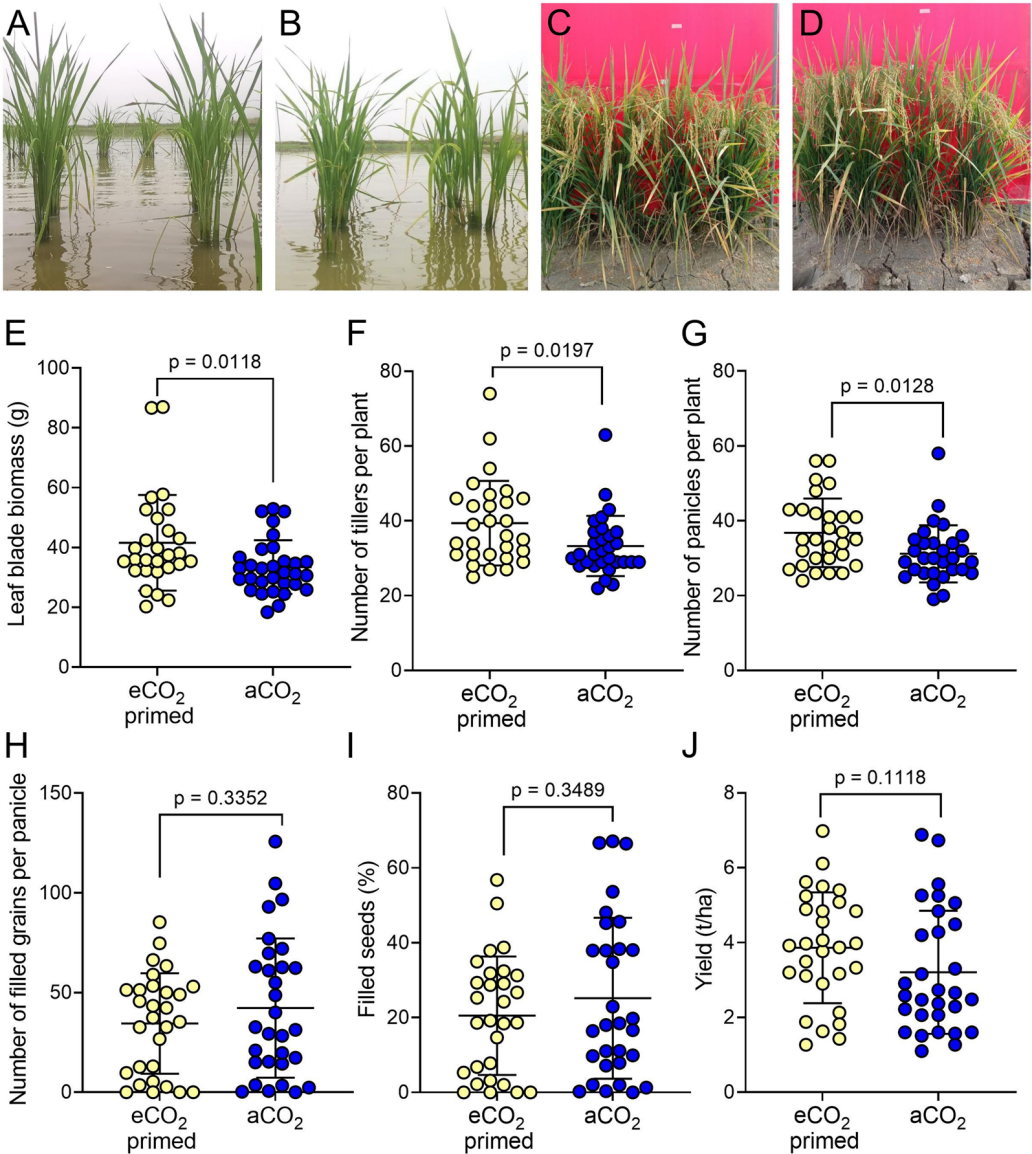
Mycelium-derived eCO_2_ primed plants grown in paddy conditions show increased yield capacity. **A** eCO_2_-primed and **B** aCO_2_ MR219 plants in the paddy at 44 DAS. **C** eCO_2_-primed and **D** aCO_2_ MR219 plants in the paddy at 120 DAS. **E**–**J** For plants harvested at 127 DAS, leaf blade biomass (**E**), number of tillers per plant (**F**) and number of panicles per plant (**G**) are significantly higher in eCO_2_-primed plants. There is no difference in number of filled grains per panicle (**H**), percentage of seeds filled (**I**) or yield of rice grain per hectare (**J**) between the treatments. **E** Mann–Whitney U test, *p* = 0.0118, n = 29/30; **F**–**J** Unpaired t tests, *p* values as shown, n = 29/30

After 28 days in the mycelium eCO_2_ chambers, seedlings were transplanted to paddy conditions (Fig. 5A, B) and grown to maturity (Fig. 5C, D). In a similar manner to the semi-field trial, paddy grown plants after eCO_2_ priming were larger at harvest (127 DAS), with a greater final leaf blade biomass compared to aCO_2_ controls (Fig. 5E, Mann–Whitney U test, *p* = 0.0118, n = 29/30). There was an increased number of tillers (Fig. 5F, unpaired t tests, *p* = 0.0197, n = 29/30) and more panicles per plant (Fig. 5G, Mann–Whitney U test, *p* = 0.0118, n = 29/30) than controls grown in aCO_2_ chambers for the first 28 days. However, unlike the rain shelter experiment, these positive outcomes on tiller and panicle number did not translate into an increase in yield in the eCO_2_ primed plants, with no significant differences in number of filled grains per panicle (Fig. 5H, percentage of seeds which were filled (Fig. 5I), indeed these values tended to be smaller in the eCO_2_-treated plants. This compensation for increased tiller and panicle number resulted in overall estimated yield per ha being similar for both treatments (Fig. 5J, unpaired t tests, *p* = 0.1118, n = 29/30).

## Discussion

Increasing rice yield in a sustainable fashion is a major global challenge. The majority of efforts in this research area have focused on genetic improvements in a wide variety of traits, including structural features linked to yield (Xing and Zhang 2010; Siddiq and Vemireddy 2021). In this context, tillering has been of major interest. The number of tillers formed by a plant will set the maximum number of potential inflorescences, the maximum number of panicles, and thus the theoretical maximum number of seeds generated by a single plant. Whether this potential is reached will, of course, be dependent on a host of factors—notably the source capacity of the plant and the portion of resources transported to the developing seed (Chang and Zhu 2017). The results reported here provide an insight into the control of tillering in rice by an environmental signal, elevated CO_2_ level (eCO_2_). In particular, our work has shown that exposing rice seedlings to eCO_2_ for the first 24–28 days after germination promotes tillering in three different rice varieties and that, moreover, this increase in tiller number is generally maintained when the plants are transferred to ambient CO_2_ (aCO_2_) conditions.

Tillers are formed by a branching mechanism in the shoot apical meristem and the associated axillary meristems (Li et al. 2003, 2018; Wang and Li 2011). The rice embryo already has about three leaves, each with a dormant axillary meristem (Itoh et al. 2000). Each new leaf formed after germination has its own axillary meristem, thus already at a very early stage of growth rice seedlings have the potential to grow tillers. Under normal conditions, the potential for these meristems to form tillers is only revealed relatively late in the growth cycle as axillary bud growth is activated (Wang and Li 2011). The increased tillering observed in plants grown in eCO_2_ could reflect either a general promotion of growth via eCO_2_ which, as a by-product, leads to earlier release of axillary meristems to form tillers, or it could be a more direct action of eCO_2_ on the activation of axillary growth.

After a short eCO_2_ priming period an increase in tillers is clearly visible in sections across the base of our rice plants (Additional file 3: Fig. S3) and seedling growth is increased, as demonstrated by increased biomass. We therefore tend towards interpreting any increased tillering as a more indirect outcome of CO_2_ on growth, but further work analyzing exactly when and where axillary meristems are activated in the eCO_2_ plants would help clarify this question. It is also interesting to note that there was an element of cultivar-specificity in the degree of response to eCO_2_ priming with respect to tiller response. Again, whether this reflects differences in general plant growth response to elevated CO_2_, or whether there is an element of specificity in tillering response to CO_2_ awaits warrants further investigation.

Although there has been an increasing push towards modernising rice agronomy via, e.g., direct planting (Kumar and Ladha 2011; Jat et al. 2022; Su et al. 2022), much current rice production remains dependent upon traditional approaches in which rice seedlings are initially grown in nurseries prior to transplanting to the paddy. There is thus a time during early growth (3–4 weeks) when the entire rice crop is essentially concentrated in a small area. We explored the idea that exposing rice plants to eCO_2_ for this short phase of crop growth might lead to a similar phenotype of increased growth and tillering to increase the rice yield potential in the mature crop. By transplanting eCO_2_ primed seedlings to field or semi-field conditions, we investigated the outcome on actual crop yield.

The first challenge was to devise a method of providing elevated CO_2_ to rice seedlings outside of conventional controlled environment chambers. To do this we explored two routes: fermentation of sugars via baker’s yeast and respiration via fungal mycelium growing on lignocellulosic waste. Both approaches proved effective at elevating local CO_2_ concentrations, generating CO_2_ levels in excess of 1000 ppm. Yeast fermentation provided a more consistent level of eCO_2_, whereas fungal respiration displayed a strong diurnal rhythm, with high levels achieved during the night and levels falling towards (but not reaching) aCO_2_ levels during the day (suggesting that the CO_2_ effect may not be via photosynthesis). Despite these differences in eCO_2_ profile, a comparable response in terms of increased tillering and growth was observed. These data suggest that the response does not require a steady high level of CO_2_ and fit more to an interpretation of integrated elevated CO_2_ over a time period of a few weeks being sufficient to elicit a response of increased tillering and growth in rice seedlings. These data are consistent with previous observations made by Jitla et al. (1997) who reported an increase in seedling tillering and growth rate when germinated in high CO_2_, and a higher rice tiller number and grain yield (g plant^−1^) in controlled growth chambers provided with elevated CO_2_ from 15 days after sowing. Our data show that this early promotion of tillering in responsive cultivars is maintained as the plants grow to maturity under ambient CO_2_ level, both in field and semi-field conditions. A difference was observed, however, between our field and semi-field experiments in the extent to which the theoretical increase in yield capacity was actually translated into yield.

In semi-field conditions, with plants transferred to large containers under a rain shelter after eCO_2_ priming, we saw a significant increase in final yield. A higher number of filled grains per panicle, a higher percentage of seeds filled, and improved grain size (1000 grain weight) drove this increase. In contrast, although the plants grown to maturity in paddy field conditions after mycelium eCO_2_ priming were significantly larger than the controls, with an increased number of tillers, panicles and leaf blade biomass, this did not translate into increased yield. Notably, the proportion of seeds which were filled was less than control, i.e. the capacity for increased yield was there but not exploited by the plants.

The reasons for this are potentially complex. First of all, the plants were grown using standard agronomic practice in the region, using normal recommended supplements of N and P. It is thus possible that although the treatment led to increased sink potential, the source activity was insufficient to exploit this potential. Supplying increased fertilizer might remedy this, but obviously would lead to extra costs (economic and environmental). It should also be noted that our exploratory field experiments were heavily impacted by the recent pandemic, with access to field sites being intermittently highly restricted. Consequently, the paddy field trial was performed towards the end of the standard growing period, leading to a noticeably high level of insect pest damage. It is also worth noting that growth in elevated CO_2_ has been reported to inhibit secondary metabolism, thus limiting crop resistance to attack (Bazinet et al. 2022). Thus, there are biotic and abiotic factors which are likely to have impacted the final stage of crop growth. Now that field access is fully open post-pandemic, repeating the field experiment with or without increased nutrient supply would be of interest. The data from our semi-field experiments clearly indicate that the increased sink capacity via increased tillering and panicle formation after CO_2_ priming can be exploited by the rice plants, but achievement of this potential in the field may require increased inputs to the system.

For the exploratory field experiments, we investigated using fungal mycelium to generate CO_2_ to promote early rice growth. The reasoning behind this was to explore whether the use of commercially important fungi (oyster mushrooms) might provide farmers with an added income stream, thus adding to economic benefits derived from increased rice yield. In addition, the fungus was grown on lignocellulosic waste products which are plentiful and inexpensive in Malaysia. The fungus yields two harvests of fruiting bodies during the priming process, which provides an additional potential income. Moreover, the hyphae/cellulosic waste after priming can be sold as a product in the form of compost. Obviously, there are costs associated with building and running the chambers used in this study to prime the rice seedlings, so capital expenditure is required to implement the system. Further research to explore the use of eCO_2_ priming systems in field conditions would enable a better assessment of the application of the process in a range of rice-growing areas with different agronomic challenges and economic conditions.

## Conclusion

In conclusion, this work identifies an environmental trigger (CO_2_) which can be used to manipulate tiller formation in a range of rice cultivars. We propose and demonstrate a mechanism by which this finding can be translated to the field situation to increase yield potential, an approach which may be of interest to a wide spectrum of growers using traditional methods to grow rice.

## Supplementary Information

**Additional file 1: Fig. S1.** Yeast eCO_2_ propagator design and performance. **A** Diagram of eCO_2_ (left) and eCO_2_ (right) propagators. **B** CO_2_ concentration in aCO_2_ and yeast eCO_2_ propagators.

**Additional file 2: Fig. S2.** Mycelium eCO_2_ chamber design and performance. **A** Diagram of aCO_2_ (top) and eCO_2_ (bottom) chamber. Bottom section is open to show interior. The aCO_2_ chamber is identical inside except for the absence of mycelium bags. **B** CO_2_ concentration in aCO_2_ and mycelium driven eCO_2_ chambers showing the diurnal nature of the amplified CO_2_.

**Additional file 3: Fig. S3.** Sections through the base of aCO_2_ and eCO_2_ grown IR64 plants 28 DAS. Stems are cut 1 cm above the root/stem boundary to show developing tillers in the transverse section. eCO_2_ grown plants **A**, **C**, **E** have more tillers than aCO_2_ plants **B**, **D**, **F** unpaired t test, *p* = 0.034, n = 3. The main culm is marked ‘M’, developing tillers are marked with a red dot. Number of tillers marked in top right corner. Scale bar = 2 mm.

## Data Availability

The datasets used and/or analysed during the current study are available from the corresponding author on reasonable request.

## Abbreviations

aCO_2_: Ambient CO_2_
eCO_2_: Elevated CO_2_
MARDI: Malaysian Agricultural Research and Development Institute
DAS: Days after sowing
