# Supplementary figures and images for: Elevated CO2 Priming as a Sustainable Approach to Increasing Rice Tiller Number and Yield Potential
Source: Rice (N Y). 2023 Mar 22;16:16. doi: 10.1186/s12284-023-00629-0 (PMC10033790; doi:10.1186/s12284-023-00629-0)

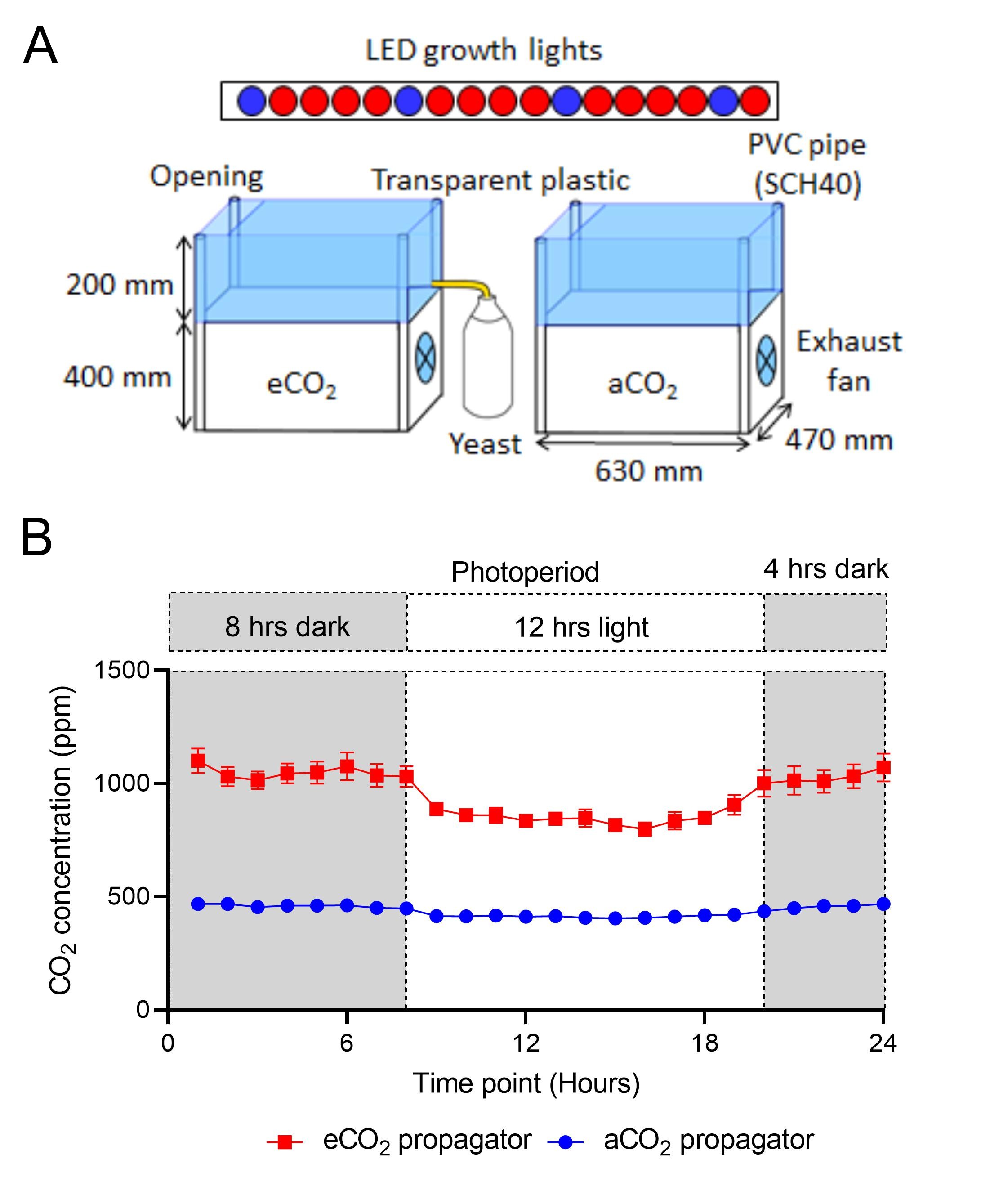

Supplement: Supplementary file 1 — Additional file 1: Fig. S1. Yeast eCO2 propagator design and performance. A Diagram of eCO2 (left) and eCO2 (right) propagators. B CO2 concentration in aCO2 and yeast eCO2 propagators. [file 12284_2023_629_MOESM1_ESM.jpg]

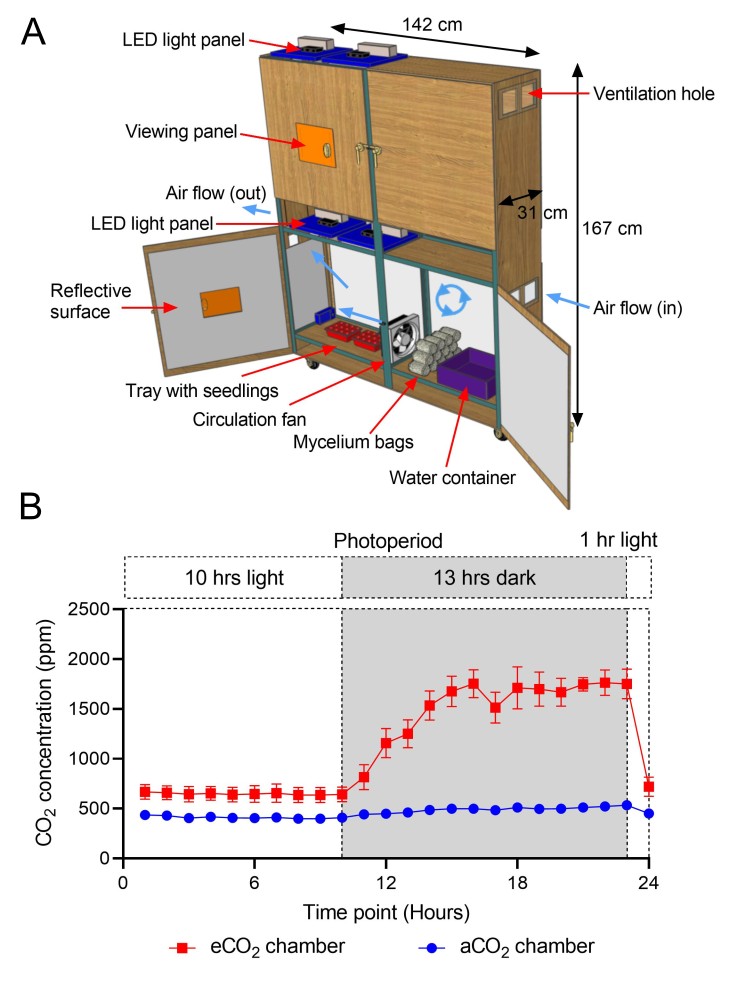

Supplement: Supplementary file 2 — Additional file 2: Fig. S2. Mycelium eCO2 chamber design and performance. A Diagram of aCO2 (top) and eCO2 (bottom) chamber. Bottom section is open to show interior. The aCO2 chamber is identical inside except for the absence of mycelium bags. B CO2 concentration in aCO2 and mycelium driven eCO2 chambers showing the diurnal nature of the amplified CO2. [file 12284_2023_629_MOESM2_ESM.jpg]

eCO<sub>2</sub>

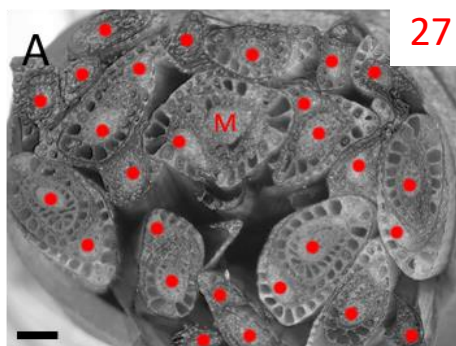

aCO<sub>2</sub>

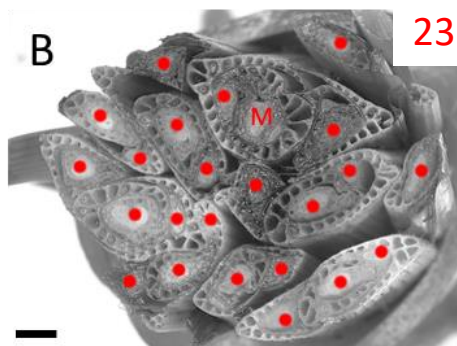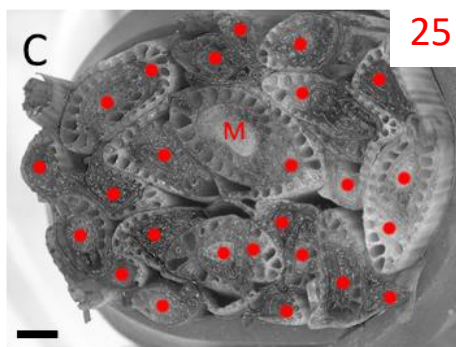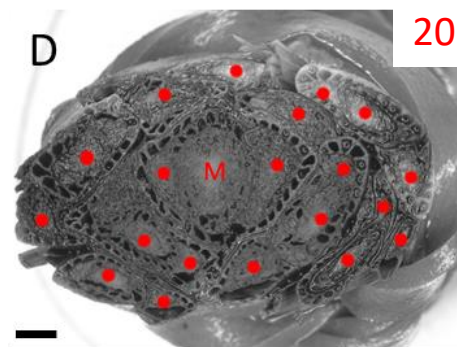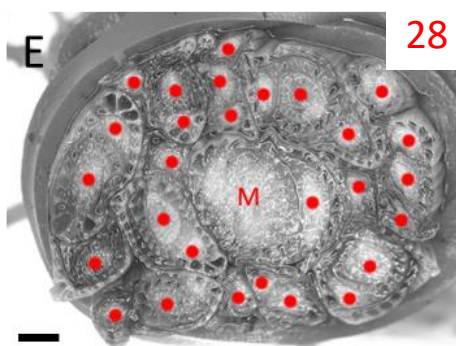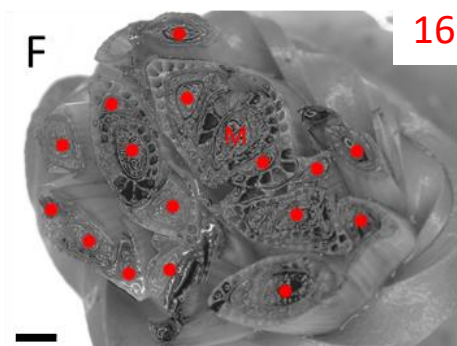

Supplement: Supplementary file 3 — Additional file 3: Fig. S3. Sections through the base of aCO2 and eCO2 grown IR64 plants 28 DAS. Stems are cut 1 cm above the root/stem boundary to show developing tillers in the transverse section. eCO2 grown plants A, C, E have more tillers than aCO2 plants B, D, F unpaired t test, p = 0.034, n = 3. The main culm is marked ‘M’, developing tillers are marked with a red dot. Number of tillers marked in top right corner. Scale bar = 2 mm. [file 12284_2023_629_MOESM3_ESM.pdf]
